# Long-distance passive dispersal in microscopic aquatic animals

**DOI:** 10.1186/s40462-019-0155-7

**Published:** 2019-03-26

**Authors:** Diego Fontaneto

**Affiliations:** National Research Council of Italy, Water Research Institute, Largo Tonolli 50, 28922 Verbania Pallanza, Italy

**Keywords:** Biogeography, Cosmopolitism, Dormancy, Meiofauna, Nematoda, Phylogeography, Rotifera, Tardigrada

## Abstract

Given their dormancy capability (long-term resistant stages) and their ability to colonise and reproduce, microscopic aquatic animals have been suggested having cosmopolitan distribution. Their dormant stages may be continuously moved by mobile elements through the entire planet to any suitable habitat, preventing the formation of biogeographical patterns. In this review, I will go through the evidence we have on the most common microscopic aquatic animals, namely nematodes, rotifers, and tardigrades, for each of the assumptions allowing long-distance dispersal (dormancy, viability, and reproduction) and all the evidence we have for transportation, directly from surveys of dispersing stages, and indirectly from the outcome of successful dispersal in biogeographical and phylogeographical studies. The current knowledge reveals biogeographical patterns also for microscopic organisms, with species-specific differences in ecological features that make some taxa indeed cosmopolitan with the potential for long-distance dispersal, but others with restricted geographic distributions.

## Introduction

Microscopic animals are generally assumed to be extremely widespread, up to the level of not showing any biogeographical pattern: the first European expeditions to remote areas brought back home surprising new groups of plants and large animals, whereas all the microscopic organisms collected during the same expeditions resembled the species that were already known in Europe [1]. Such empirical scenario of widespread distribution was found across different taxa and at different spatial scales. Among the different rationales that tried to explain the pattern of cosmopolitism in microscopic animals the most known remains nowadays the ‘everything is everywhere’ or the ubiquity hypothesis [2–5]. The discussion on the lack of biogeography for microscopic organisms focused originally on prokaryotes and unicellular eukaryotes, to then extend also to all microscopic organisms below one or two millimetres in body length, including microscopic multicellular animals [1, 6]. The ubiquity hypothesis for microscopic aquatic animals holds true for continental [7] as well as for marine species [8], for which it is known as the meiofauna paradox: microscopic benthic organisms with little mobility seem to be cosmopolitan, contrary to their lack of dispersal ability and their lack of planktonic larval stages [9].

The inclusion of microscopic animals in the discussion on the ubiquity hypothesis makes comparisons with larger organisms more obvious: microscopic and macroscopic animals have common physiology, similar ecology, and shared evolutionary trajectories [10]. Yet, biogeographical patterns seem to be different between microscopic and large animals, because of the differences in size and the ecological consequences of being microscopic [1, 6].

Three assumptions should be met in order to allow long-distance dispersal in microscopic aquatic animals: dormancy capability, long-term resistance of dormant stages, and ability to colonise and reproduce quickly. Here I review these assumptions and the direct and indirect evidence we have for long-distance dispersal. The review is not meant to be exhaustive on each of the topics, but is designed to introduce the subject and its relevance for our general understanding of movement ecology and biogeography of microscopic aquatic animals, focusing on nematodes, rotifers, and tardigrades, the most notorious microscopic animals with high potentials for long-distance dispersal.

## Review

Microscopic aquatic animals, smaller than one or two millimetres [5], are small by definition: they cannot be easily seen with a naked eye and they are considered ecologically different from the large, macroscopic organisms that we see around us [1, 9]. One of the main consequences of being microscopic for aquatic animals is that the ratio between volume and surface of the organism is so small that when the animal is taken out of water it may be unable to retain its internal liquids, which would evaporate very quickly unless some sort of protection is present [11]. The volume to surface ratio may not usually be a problem for an unprotected microscopic animal that lives in osmotically and chemically stable, permanently hydrated habitats, such as large lakes and open oceans. Such animal will have very small or almost no probability of encountering desiccation problems. Moreover, some animals, especially arthropods, have protective integuments to contrast such rapid desiccation. Yet, several microscopic animals with no thick protective integuments live in temporary water bodies that desiccate frequently, for example shallow ponds, bogs, small puddles, intermittent rivers, cryoconites, rock pools, salt marshes, etc. Some microscopic aquatic animals even adapted to live in the thin and ephemeral water layers surrounding mosses, lichens, and soils, in what is called a limno-terrestrial habitat: an aquatic microscopic environment (the ‘limno’ part) in an otherwise terrestrial ecosystem. The most common and abundant animals living in these habitats, especially in continental freshwater settings, are nematodes, rotifers, and tardigrades, a community of microscopic animals collectively known as meiofauna [8, 9]. Other microscopic aquatic animals exist and are part of the meiofauna, e.g. gastrotrichs, loriciferans, kinorhynchs, etc., but they do not have the dormancy capabilities of nematodes, rotifers, and tardigrades that make them suitable candidates for long-distance transportation, dispersal and cosmopolitan distribution [12].

The three animal groups included in this review belong to different evolutionary lineages in the metazoan tree of life [10], but they all share small body size, usually much less than 1 mm, together with the ability to survive lack of water through dormancy [13, 14] and other peculiar features that will be introduced in this review. Overall, more than size in itself, the consequences of small size are relevant for dispersal abilities [11, 15], and these peculiar ecological features will be the subject of the first part of the review.

### Dormancy

Nematodes, rotifers, and tardigrades independently evolved adaptations to survive adverse periods without water [16–19]: as soon as conditions are not favourable with liquid water becoming unavailable because of evaporation or of freezing, some animals are able to produce dormant stages to allow the following generation to recover a viable population when liquid water becomes available again [16]. Such dormant stages are known as resting eggs in rotifers and in tardigrades [20, 21], and eggs in nematodes [22]. They are dormant embryos and not actual eggs [23], and they are produced both by parthenogenesis and by sexual reproduction. There is an ample literature on the triggers that drive the production of resting stages, and also on the mechanisms that are put in place to maximise the efficacy of such stages to maintain viable populations through bet-hedging strategies, especially for microscopic zooplankton animals [24]. The production of dormant stages that accumulate at the bottom of an ephemeral water body is also considered the main mechanism that structures community and population dynamics for microscopic animals with high genetic differentiation at the local and landscape level [25], in what was named monopolisation hypothesis [26]. These communities, structured by the interplay between the buffering effect of dormant stages acting against new colonisers and the dispersal of dormant propagules from other populations [27, 28], are one of the most studied examples within the metacommunity framework [29, 30]. The research output of community ecology connected to dispersal in microscopic aquatic animals is highly productive, but will be only marginally considered in this review, given that it involves local or landscape-level settings, and not proper long-distance dispersal across continents.

Microscopic animals can withstand lack of liquid water through other mechanisms, not only through the production of dormant embryos: bdelloid rotifers and tardigrades can simply contract into a ‘tun’ shape and nematodes can coil, losing most if not all of their internal water, halting any metabolic activity, and remaining in this dormant condition until water becomes available again [31, 32]. The physiological mechanisms allowing these animals to remain viable while desiccated during dormancy are still under study, and are thought to involve protecting molecules such as sugars, late embryogenesis abundant proteins, antioxidants, etc. [33–35], in addition to the ability to recover their broken DNA when rehydrated [36]. Two main scenarios have been identified on the effect of dormancy on life span: either metabolism is completely halted and animals do not age during dormancy (the ‘sleeping beauty’ model) or some stress acts on the animals, which do age slowly while dormant (the ‘picture of Dorian Gray’ model) [37]. Such differences have obvious consequences for long-term viability, which can be much longer for groups that follow the ‘sleeping beauty’ model [38]. Rotifers and tardigrades seem to follow the ‘sleeping beauty’ model, whereas nematodes seem to follow the ‘picture of Dorian Gray’ model [37–40].

Dormancy may be attained several times during the life span of an organism: for example, meiofauna living on a lichen patch on a tree trunk at temperate latitudes may desiccate every day under the sun in the morning and resume activity every evening when moisture levels increase again with enough water in the thin layers surrounding the lichen to allow the animals to move and feed. Given that the life span of these animals is usually more than a month [20, 22, 41, 42], they may experience several tens of cycles of desiccation and resurrection during their life. Desiccated dormant animals can be easily moved by wind through the landscape, and this is considered the main mechanism that strictly asexual bdelloid rotifers use to survive their arm-race against fungal parasites in a hide-and-seek continuous movement to new lichen patches temporarily without parasites [43].

One caveat about generalisations on dormancy is that interspecific variation in survival during dormancy is known in nematodes [44, 45], rotifers [46–48], and tardigrades [49–51]. Thus, even if these three groups of microscopic aquatic animals indeed have the capacity to survive adverse conditions through dormancy, there is a degree of species-specific survival capabilities while dormant. Some species are indeed known not to survive dormancy at all [21, 22, 52].

### Long-term viability

The simple fact of being small forces microscopic aquatic animals to develop mechanisms to survive lack of water, either through the production of dormant propagules such as protected resting embryos that will resume development after rehydration, or through the dormancy capability of the animals themselves, able to enter a desiccated stage at any time of their life [16]. For dormant stages of any type to be successful in long-distance transportation and successful dispersal, they have to remain under dormancy for long time, enough at least to be moved by mobile vectors across continents, and then to be able to recover and ‘resurrect’ [16]. Dormant stages have also to survive adverse conditions other than desiccation, such as high and low temperatures, oxidative stress, UV radiation, digestive enzymes, etc. [53–55]; at the same time, they still need to react to external stimuli, in order to hatch when environmental conditions are favourable [19, 56, 57].

The potential maximum length of long-term viability of dormant stages is unknown and not easy to test; yet, there is evidence that such survival can be extremely long. Dormant animals of the limno-terrestrial meiofauna could be recovered after more than 10 years of artificial dry storage of lichens in museum collections [58]. The longest unambiguous survival times for adult dormant animals are those of tardigrades from dry mosses, which are up to 20 years [50, 59–61], and those of nematodes from sediments, even more than 30 years [22, 62, 63].

Regarding resting embryos, the oldest viable resting eggs of rotifers that have been successfully hatched from sediments were older than 100 years and belonged to two species of the genus *Brachionus*, *B. calyciflorus* and *B. plicatilis* [64, 65]. Resting stages of other small aquatic animals like copepods are known to remain viable even longer, for a few centuries [66, 67]. Thus, the potential for long-term viability is an actual feature of meiofauna, both for resting stages and for dormant animals.

As a consequence of their dormancy capabilities, the three main groups of the meiofauna, nematodes, rotifers, and tardigrades, are able to cope with the extreme conditions of the most hostile places for life on Earth, being for example the only metazoans thriving in polar deserts [68–72]. As a by-product of their resistance, they have been shown to remain viable also when exposed to environmental conditions that they likely never experienced during their evolution, such as those of the outer space [73–76].

Another ecological consequence of long-term viability during dormancy for microscopic aquatic animals is that they can be found even outside of their ecological niche [77]. For large animals, because of dispersal limitations, the realised niche (where the organism is actually found) is geographically nested within the fundamental niche (the areas where the organism could survive) [78]: for example, a mammal or a beetle living in Eurasiatic temperate forests may be absent from the North-American equivalent forests not because of habitat unsuitability, but simply because it never arrived there. For microscopic aquatic animals, one can speculate that if the assumption of reduced dispersal limitation because of dormancy resistance is true at least for some species, the realised niche (where such species are found) can in principle expand well beyond the fundamental niche (where the species survives): sink populations, where population growth rate is negative, can be maintained by propagules coming from large source populations placed in suitable areas [78]. This pattern is known for other organisms with dormant dispersing propagules, such as thermophilic bacteria in cold soils [79], or in the continuous recruitment of marine algae in unstable environments from more stable source populations [80]. The discrepancy between a large fundamental niche and a smaller realised niche in large organisms makes them considered as “alien species” when displaced elsewhere by human activities [81]. If microscopic aquatic animals are already cosmopolitan, in principle, no alien species should exist, because they are already present wherever human activities may bring them: the issue of alien species in nematodes, rotifers, and tardigrades will be discussed later, when dealing with biogeographical evidence of long-distance dispersal.

### Parthenogenesis

Reproductive capabilities do not matter for long-distance transportation and dispersal per se, but will affect the possibility of the propagule to effectively colonise the new place where it lands. Nematodes, rotifers, and tardigrades share dormancy capabilities and the possibility of parthenogenetic reproduction: a single dormant female or a dormant embryo can start a new population after recovering from dormancy. Different groups have different parthenogenetic strategies. Bdelloid rotifers are strictly asexual, monogonont rotifers reproduce by cyclical parthenogenesis [82], tardigrades and nematodes have different degrees of parthenogenesis and hermaphroditism between species [22, 83] and sometimes even between different populations within species depending on the habitat [84]. Thus, almost all species of nematodes, rotifers, and tardigrades have the potential to rapidly develop a new population, even if only one single propagule arrives through long-distance transportation.

### Evidence of long-distance dispersal

Microscopic aquatic animals like nematodes, rotifers, and tardigrades fulfil the assumptions of being small, surviving in dormant state, and reproducing rapidly when conditions return suitable. Because of these features, they have been assumed to be able to disperse passively across the globe [4, 5, 85, 86]. Long-distance successful dispersal, defined as low frequency dispersal events in the tail of the dispersal kernel [87], does not need to be a common phenomenon, and even a relatively small number of successful propagules can be enough to allow these animals to be geographically widespread [88]. Yet, do we have evidence that indeed they do disperse? Direct studies of transportation and dispersal through capture, marking, and recapturing techniques [89] are impossible for meiofauna. So, how can people discuss about their long-distance dispersal? We have two main lines of evidence: on one hand, there is direct evidence that microscopic animals can passively move across the landscape; on the other hand, there is indirect evidence of actual cosmopolitism from biogeographical studies that use a morphological approach in species identification, and phylogeographical studies that analyse the geographic distribution of DNA sequences within species. Here, I provide a brief overview of such direct and indirect evidence.

### Direct evidence

Microscopic animals can be passively dispersed from one place to another by mobile vectors such as larger animals that are able to actively move across the landscape or by wind and water [90]. Dispersal of dormant stages allows meiofaunal organisms to escape unsuitable environmental conditions, to temporarily avoid competition and parasitism, and thus to move to distant areas and new habitats [91].

Transportation and dispersal by a mobile element such as an animal vector is a known mechanism in movement ecology and is commonly called phoresy [92]. Phoresy is a temporary symbiotic interaction between one organism and a host for the purpose of travel. Known examples of phoresy involve ticks, mites, and pseudoscorpions attaching to larger arthropods and to vertebrates and has been described even in fossils from several million of year ago [92]. Microscopic aquatic animals are also known to hitchhike with phoretic relationships [93], but the phenomenon is not so common.

Microscopic aquatic animals have been found dispersing by epizoochory, externally attached to a variety of larger animals [94]: for example, on earthworms [95], beetles [96], water bugs [97], fish [98], treefrogs [99], flying foxes [100], wild boars [101], nutrias [102], and even humans [103, 104]. Endozoochory, the transportation through the digestive gut of large animals, is also possible for microscopic aquatic animals, and viable invertebrates have been found in faeces of vertebrates [105, 106]. These examples are relevant to show that meiofauna can be passively dispersed by mobile elements across the landscape [107]. Yet, such mobile elements (except for humans) cannot be the cause of the widespread distribution of several species of nematodes, rotifers, and tardigrades, because the hosts themselves are not so widely distributed across continents and their distributions are indeed limited by barriers to active dispersal [108]. Thus, notwithstanding the relevant effect of phoresy and endozoochory by macroscopic animals for community ecology at the landscape level, only limited effects can be expected for long-distance dispersal and biogeography of meiofauna.

The only mobile animals that could allow meiofauna to disperse across large distances are migratory birds [109]: indeed microscopic animals have been found associated to birds. Since Darwin’s time researchers placed feet of ducks in water and performed other experiments to find viable microscopic animals attached to or in the gut of migratory birds [7, 110–112]. Yet, dispersal of meiofauna through birds may not be as effective as for seeds of plants [105], and can be considered plausible only for those microscopic animals that live in shallow water or in other habitats that are frequently visited by birds. This can be true for marshes and ponds [110], rock pools [113], littoral sediments [114], and even vegetation eaten by birds [109], but birds cannot be considered a suitable mobile element for meiofauna living for example in vertical lichen patches on tree trunks or in other habitats not visited by birds.

The most plausible mobile elements that can disperse dormant propagules of all microscopic aquatic animals regardless of the habitat are wind for terrestrial meiofauna and water for marine meiofauna. Colonisation experiments of mesocosms designed to selectively exclude different mobile elements indeed found meiofauna when allowing only wind as a carrier, and also demonstrated that the effect of wind was much stronger than that of phoretic animals in dispersing propagules across the landscape [115–119]. Meiofauna can be found dispersing through wind and water [120–126], and dormant propagules of meiofauna can be found in windsocks [127], on sticky traps [128], or in desert dust storms [129]. It is true that the number of dormant propagules found in direct sampling of wind and air is usually low, but even a small number of successful dormant propagules is anyway predicted to allow for effective long-distance dispersal, especially if those propagules can rapidly develop large populations via parthenogenesis [87, 125, 130, 131].

A mechanism for trans-oceanic dispersal between continents, demonstrated at least for nematodes, is rafting: some nematodes are able to move across long distances inside rhizome fragments that are dispersed by seawater [132]. The same holds true also within the marine environment, where benthic meiofauna can be found in the water column [133, 134] and in suspended sediment traps physically disconnected from the bottom [135].

Overall, our current understanding is that dormant propagules of nematodes, rotifers, and tardigrades have the potential for long-distance dispersal and propagules for some species have been found to be transported and dispersed globally. Nevertheless, a quantification of the role of such potential long-distance dispersal on the biogeography of microscopic aquatic animals is unavailable, and the relative role of animal-mediated, wind-mediated, and water-mediated effect on dispersal is still debated. Unfortunately, no quantification of long-distance transportation and dispersal is possible and no quantitative modeling has been developed yet to address such issue, contrary to what has been successfully done for seed dispersal in plants [136, 137].

### Biogeography

The widespread distribution of microscopic organisms was the first evidence to indirectly suggest the possibility of long-distance dispersal, within the rationale of the ‘everything is everywhere’ or ubiquity hypothesis [5]. Indeed, several species of nematodes, rotifers, and tardigrades are known to occur in different continents. On the other hand, there is also a long list of species from these phyla with restricted distribution, making any generalisation on their biogeography ambiguous.

Nematodes with limited dispersal abilities have biogeographical patterns even at the genus level, with several genera having different species in different continents [138]. Different continents have a different composition in genera and families with consistent differences [12]. Differences in species composition between nematode communities correlate with geographic distances across continental scales [139]. Thus, for nematodes, biogeography seems to exist and cosmopolitism is an exception.

In rotifers, most species are considered cosmopolitan [140] and surveys usually find a large proportion of cosmopolitan species [141, 142], but endemic species exist too [143]. In addition to a large number of cosmopolitan species, a large representation of the global diversity is usually found in local samples: any single temperate or tropical water body is expected to host between 150 and 250 species, that is up to 12% of global worldwide diversity found locally in one single water body [144]. Rotifer biogeography is a complicated issue: biogeographical patterns exist, but are hard to see and occur mostly at a continental scale [145, 146].

In tardigrades, the distribution of genera and families is different between landmasses originating from Laurasia and Gondwana [147–149]. Of the 64 limno-terrestrial genera of tardigrades, 11 are considered endemic at continental level [12], and continental endemism at the species level can be up to 60% [148]. In addition, species of the genus *Mopsechiniscus* follows the geological division of Gondwanan landmasses [150]. For tardigrades, biogeography indeed makes sense.

Among the main problems in assessing biogeographical patterns for all organisms, large and small, are lack of data and sampling bias [151, 152]. Our knowledge on the distribution of microscopic aquatic animals suffers from such issues even more than for larger organisms [145, 153]: for example, after compiling all published records on monogonont rotifers from over 1800 published papers across more than 30 years, the most significant predictor of species richness remained sampling effort, obscuring a negligible effect of spatial and environmental drivers [146]. The same lack of data and sampling bias hold true also for tardigrades [154].

The probability of finding endemic species in meiofauna negatively correlates with species-specific dispersal abilities [155]. In general, rotifers seem to have low endemism, whereas nematodes and tardigrades seem to have clear biogeographical patterns.

An interesting support against the claim of cosmopolitism in microscopic aquatic animals is the presence of alien species: they exist also for microscopic aquatic animals, even in rotifers [156–160], suggesting that at least some species were not able to colonise some areas before they were introduced recently by human activities.

### Phylogeography

Phylogeographic structures in small organisms can be very complex, with interacting effects of local adaptation buffering against newcomers and the action of geographically structured gene flow between diverse lineages from different geographic areas [161, 162]. Priority effects, founder events, and genetic bottlenecks are common scenarios found in spatially explicit surveys of genetic diversity for nematodes [163–165], rotifers [166], and tardigrades [167], both in freshwater and in marine water bodies.

When using DNA sequence data to analyse spatial structure in the distribution of microscopic aquatic animals, researchers realised that our understanding of diversity was highly biased, with several species clearly indicated as independently evolving entities that could not be identified as separated with a morphological approach [168]. A large number of cryptic species is continuously discovered in almost all analysed meiofaunal taxa [169–172]. Thus, if species that were considered cosmopolitan are in reality complexes of geographically restricted species, the biogeographical inference based on morphology could be misled by inappropriate identification of taxonomic units. Yet, again, also in the case of cryptic species complexes, there are examples where several of the actual species of the complex came out as widespread [173] and examples where each of the species in the complex had geographically restricted distribution.

In nematodes, both long-distance dispersal and restricted gene flow was found using DNA sequence information [164, 174].

In rotifers, the most studied species complex, *Brachionus plicatilis*, for which hundreds of populations have been sequenced, is composed in reality by at least 15 different species, but most of them have cosmopolitan distributions [175, 176], even if often with local phylogeographic structures [177–180]. The same global distribution but with local genetic structure is found in bdelloid rotifers too [181, 182].

In tardigrades, geographically restricted species within a species complex, previously considered cosmopolitan but now known to be composed of several taxa with limited distribution were found [170], for example in the marine tardigrade *Echiniscoides sigismundi*, where populations display extreme subdivision and contain deeply divergent lineages that are fully or nearly restricted to single sampling sites [167, 183, 184]. *Echiniscus testudo* seems to be a single species and not a complex, but indeed very widespread, if not cosmopolitan [61, 185]. Antarctic tardigrades seem to have several narrowly distributed species [186].

Most of the studies using DNA sequence data, similarly to the studies on biogeography, reveal little or no connection between geographic and genetic distances between populations, but provide evidence of limited distributions, potentially linked to dispersal limitation, together with some examples of truly cosmopolitan distributions. Thus, until now, not even quantitative methods from population genetics in spatially explicit contexts allowed generalisations and quantifications of the role of long-distance dispersal in shaping the distribution of nematodes, rotifers, and tardigrades.

## Conclusions

The whole range of biogeographic patterns seems to be present in nematodes, rotifers and tardigrades, from actual cosmopolitan species through long-distance dispersal, according to the ‘everything is everywhere’ hypothesis [5], to endemic ones, more in line with the moderate endemicity hypothesis [187], similar to what is now known also in other microscopic organisms [188, 189], dismissing the general view that microscopic aquatic organisms are uninteresting for biogeography because they are all cosmopolitan [1].

Such broad diversity in biogeographical, and consequently phylogeographical, patterns seem to be related to species-specific dormancy capability: similar to what happens in many terrestrial species of plants [190], species-specific limited dormancy and dispersal capacity can restrict the distribution of some species of microscopic aquatic animals. Species with higher dispersal potential exhibit greater viability of dormant stages [191, 192]. The importance of dispersal differs greatly among and within species, depending on their life histories [7, 193]. Some species of microscopic aquatic animals are indeed cosmopolitan, but not all [1].

It is true that microscopic aquatic animals have incredible dormancy capabilities [40] in principle allowing them to be passively dispersed, but their actual dispersal depends on several factors, both internal (e.g. species-specific dormancy strategy, long-term viability, etc.) and external (e.g. habitat type, mobile element, etc.). Because of species-specific differences in dormancy, potential for long-distance dispersal, biogeographical and phylogeographical patterns, and in spatial structure, the study of dispersal and biogeography of microscopic aquatic animals, contrary to being uninteresting, may become highly relevant to support or refute the generality of the patterns and processes assumed for larger organisms [193, 194]. Moreover, because of their small size, the use of such animals as biogeographical models may open new frontiers in biodiversity and biogeography, even with experimental biogeography, which will be impossible to achieve with larger organisms [193–195].
